# Piloting an Innovative Concept of e–Mental Health and mHealth Workshops With Medical Students Using a Participatory Co-design Approach and App Prototyping: Case Study

**DOI:** 10.2196/32017

**Published:** 2022-01-10

**Authors:** Melina Dederichs, Felix Jan Nitsch, Jennifer Apolinário-Hagen

**Affiliations:** 1 Institute of Occupational, Social and Environmental Medicine Centre for Health and Society, Faculty of Medicine Heinrich Heine University Düsseldorf Düsseldorf Germany; 2 Comparative Psychology Institute for Experimental Psychology Heinrich-Heine-University Düsseldorf Düsseldorf Germany

**Keywords:** participatory design, co-design, mHealth, medical student, eHealth, medical education, mental health, mobile phone

## Abstract

**Background:**

Medical students show low levels of e–mental health literacy. Moreover, there is a high prevalence of common mental illnesses among medical students. Mobile health (mHealth) apps can be used to maintain and promote medical students’ well-being. To date, the potential of mHealth apps for promoting mental health among medical students is largely untapped because they seem to lack familiarity with mHealth. In addition, little is known about medical students’ preferences regarding mHealth apps for mental health promotion. There is a need for guidance on how to promote competence-based learning on mHealth apps in medical education.

**Objective:**

The aim of this case study is to pilot an innovative concept for an educative workshop following a participatory co-design approach and to explore medical students’ preferences and ideas for mHealth apps through the design of a hypothetical prototype.

**Methods:**

We conducted a face-to-face co-design workshop within an elective subject with 26 participants enrolled at a medical school in Germany on 5 consecutive days in early March 2020. The aim of the workshop was to apply the knowledge acquired from the lessons on e–mental health and mHealth app development. Activities during the workshop included group work, plenary discussions, storyboarding, developing personas (prototypical users), and designing prototypes of mHealth apps. The workshop was documented in written and digitalized form with the students’ permission.

**Results:**

The participants’ feedback suggests that the co-design workshop was well-received. The medical students presented a variety of ideas for the design of mHealth apps. Among the common themes that all groups highlighted in their prototypes were personalization, data security, and the importance of scientific evaluation.

**Conclusions:**

Overall, this case study indicates the feasibility and acceptance of a participatory design workshop for medical students. The students made suggestions for improvements at future workshops (eg, use of free prototype software, shift to e-learning, and more time for group work). Our results can be (and have already been) used as a starting point for future co-design workshops to promote competence-based collaborative learning on digital health topics in medical education.

## Introduction

### Background: Medical Students and the Potential of Mobile Health Apps

Medical students’ poor mental health remains a worldwide challenge. Despite the high prevalence of mental illness among medical students [1], they are less likely to seek help than age-matched controls [2]. Moreover, they encounter additional hurdles when seeking help, such as fear of stigmatization or disadvantages for their prospective career [2-4]. Thus, face-to-face counseling on campus might not be the appropriate option for all students. Mobile health (mHealth) apps could help reduce the treatment gap.

Previous research suggests that digital interventions can be an effective tool to promote university students’ mental health [5]. They address several barriers such as fear of stigmatization and provide help independently of time and location [6-8]. Especially in the face of the ongoing COVID-19 pandemic, digital access to psychological support is more important than ever [9]. However, the uptake of suitable mHealth apps among college students remains relatively low [10,11]. Evidence of acceptance of mHealth apps, including mental health apps, among university students is still limited and inconclusive [5,12,13], especially regarding the subgroup of medical students [14].

A possible explanation for the low uptake of mHealth apps might be that existing mHealth apps do not reflect medical students’ needs and preferences [15]. Understanding users’ needs might help to increase the early acceptance and use of mHealth apps [16]. A further reason for the low uptake might be that medical students lack familiarity with mHealth apps. For instance, in a recent study, only 1.3% of the participating German medical and psychology students reported ever having used an mHealth app [15]. Moreover, medical students’ understanding of several aspects concerning mHealth apps (eg, terminology) is limited [17-20]. This knowledge is of great relevance for medical students, from both user and health care provider perspective. In December 2019, the German government passed the Digital Healthcare Act, which allows for the prescription of medical apps, including mental health apps [21]. As future health care providers, medical students will influence digital health care by prescribing mHealth apps to their patients [15]. Thus, they play a key role in facilitating the awareness and acceptance of mHealth apps in the general population. However, education on e–mental health and mHealth is still rare in medical curricula in Germany [22] as well as the rest of the world [23,24].

Given the relevance of the subject, educational concepts are needed to help implement mHealth in the medical curriculum.

### Goals of This Case Study

The primary aim of this case study is to describe the piloting of a novel co-design workshop on mHealth and e–mental Health at a German medical school. We sought to explore the feasibility of co-design workshops as an educational concept and asked for participants’ evaluations and suggestions for improvements regarding future co-design workshops (iterative development). Furthermore, we were interested in medical students’ ideas and preferences for prototypes of mHealth apps and their application of the theoretical insights conveyed during the workshop.

## Methods

### Participants and Setting

The participants in this case study were preclinical and clinical medical students enrolled at the medical school of Heinrich Heine University Düsseldorf (HHU) in Germany. The inclusion criteria were age ≥18 years and registration for the elective course *e–Mental Health in Medical Education* (ie, the co-design workshop). Participation in this study was voluntary and did not affect the successful completion of the course. All participants gave their informed consent and agreed that their data (eg, their feedback and ideas for mHealth apps) could be used for research purposes. The study was approved by the ethical committee of the medical faculty of the University of Düsseldorf as part of a medical education project called *Healthy Learning in Düsseldorf*, which aims to investigate and improve medical students’ mental health (study number: 4041).

### The Co-design Workshop

We conducted the co-design workshop on 5 consecutive days on site from March 2 to 6, 2020, at the Faculty of Medicine at HHU, approximately 1 week before the first COVID-19 lockdown [25] and approximately 7 months before the directory for prescriptible digital health apps (*Verzeichnis für digitale Gesundheitsanwendungen* or Digital Health Applications Directory) was thrown open to the public in Germany [26]. The duration of the workshop was 30 hours in total, delivered over 5 days (11 AM to 5 PM from Monday to Friday), and it was held during the semester holidays. The workshop comprised lectures and supervised group work. Each day was designated for a specific topic or method of intervention development with respect to e–mental health (including participatory design approaches). Different modules guided the students through the development of a rapid prototype of their own mental health app (Table 1). In all, 3 guest lecturers were involved on days 2-4 to give insights into the development of mHealth apps. We conducted focus groups on the second and third day, which have been reported elsewhere [27].

Generally, each day of the workshop was structured in 2 parts. The first part consisted of introductory lectures on eHealth and participatory design methods. Moreover, the students were shown how to identify existing mHealth apps that are safe to use and are also of high quality. During the second part, the participants were divided into smaller groups to work through relevant literature on e–mental health and to develop their own hypothetical prototype for a mental health intervention. For this, they used a range of methods grounded in participatory design, design thinking, and target population–centered approaches to intervention development (Table 1). The students could create the concept for a native app or a web-based app (web version optimized for smartphone screens). They were asked to implement and consider everything that they deemed important.

**Table 1:** Workshop contents.

Workshop day	Educational content	Activity
1	Introduction, components and types of guidance for mHealth^a^ apps, quality criteria for mHealth apps, and legal framework	Introductory lecture, group work, presentations and plenary discussions on relevant literature, legal aspects, and identification of existing mHealth apps that are safe to use and are also of high quality (MARS-G^b^ [28])
2	Acceptability and user orientation, co-design and participatory design methods, and strategies and model for designing mHealth apps	Expert lecture (building your own mHealth app and insights into a medical student’s back pain app start-up), focus groups part 1 (reported elsewhere [27]), participatory approaches: think-aloud technique, and IDEAS^c^ [29]
3	Gamification, development and adjustment of mHealth apps, and avatars	Expert lecture (assessment of avatar of a certified medical app for insomnia, *Somnio*), storyboarding, focus groups part 2 (reported elsewhere [27]), and prototyping avatars in groups
4	Acceptance-facilitating interventions, adherence-facilitation, and implementation	Expert lecture (web-based marketing), persona development, journey mapping, implementation mapping, prototyping, development of personas in groups, and mock-ups and prototypes
5	Presentations and workshop evaluation	Presentations of the mHealth app concepts, feedback questionnaires, and feedback round

^a^mHealth: mobile health.

^b^MARS-G: Mobile App Rating Scale, German version.

^c^IDEAS: Integrate, Design, Assess, and Share.

The first author (MD) and the last author (JAH) facilitated the workshop. JAH created the contents of the workshop with the support of MD. MD is a researcher and trained psychologist. JAH is a qualified psychologist with a background in medical psychology and an experienced researcher with focus on e–mental health and mHealth acceptance in different target groups, including medical students, as well as psychosocial stress research. Both facilitators conduct lectures for medical students and are involved in research on medical students’ well-being. They took turns conducting lectures; the other observed and took field notes.

### Prototypes of Apps: Knowledge Transfer

Each group, consisting of 3-7 students, focused on a different psychological problem for their hypothetical prototype of an mHealth app. Participants could choose from among the following 5 predetermined group topics, selected on the basis of their relevance for promoting mental health among medical students [1,30-32]:

- Depression and anxiety (transdiagnostic; students chose to focus on depressive symptoms)
- Stress management and subjective well-being
- Test anxiety and procrastination
- Insomnia (focus on health behavior and sleep quality)
- Psychosomatic conditions (self-management of chronic conditions; students chose to focus on gastrointestinal problems)

The medical students also had the opportunity to adapt their topic or propose other health conditions. All groups were supervised and provided with feedback during the development phase of their mHealth app.

### Data Collection and Descriptive Analysis

During the workshop, the facilitators took notes and documented the workshop with photographs. All written and designed material was collected with the permission of the participants. In addition, the participants filled out a so-called logbook with predefined tasks mirroring the contents of the workshop. The logbook was also used to document their thoughts, ideas, and progress regarding the development of their prototype. The final segment of the workshop was devoted to the group presentations of the hypothetical app concepts. The presentations were rated based on predefined evaluation criteria (Textbox 1). The groups could choose to present their hypothetical prototypes using either a digital or flip chart presentation format. Of the 5 groups, 4 (80%) chose a digital presentation format. The presentations were analyzed, where possible, based on the extended version of the Unified Theory of Acceptance and Use of Technology, (UTAUT2 [33]) which has been introduced in the workshop. UTAUT2 has been postulated as a framework to understand and predict technology uptake and use. The model comprises 4 constructs from the original model (effort expectancy, facilitating conditions, performance expectancy, and social influence [34]) as well as 3 additional constructs (habit, hedonic motivation, and price value). The UTAUT2 model has been used in different contexts such as acceptance of electronic medical records or mobile learning technology [35-39]. Here, for instance, we looked at whether the medical students’ prototypes included elements that foster hedonic motivation, such as gamification (Textbox 1). In some cases, we needed to extend the categories inductively based on the material because UTAUT2 did not provide a suitable category such as data security.

**Criteria for the evaluation of medical students’ presentations on their prototypes of mobile health apps.**

**Evaluation criteria**

- Quality of the content
- Relevance for the target group and field of action
- Overall concept and presentation: comprehensibility, rationale, and logic
- Selection of content and components (based on evidence, empiricism, etc)
- Practical transfer: strategies for dissemination and execution
- Implementation
- Manner of presentation
- Visualization

At the end of the workshop, the participants completed a brief feedback questionnaire to evaluate the workshop. They were asked 3 questions regarding their perceived learning progress during the workshop on a scale from 1 (strongly disagree) to 6 (strongly agree), and they were given the opportunity to add suggestions for improvement in free text. In addition, feedback was collected during an oral feedback round and within a standardized anonymized evaluation form for lectures at medical schools. The latter is not reported in this study. The participants’ feedback was used to make alterations and improvements for future workshops. Statistical analysis of the paper-based questionnaire data was performed using the software SPSS (version 25.0; IBM Corp).

## Results

### Sample Characteristics

In all, 26 participants (women: 17/26, 65%; men: 9/26, 35%) aged 18-30 years (mean 23.35, SD 3.73 years) took part in the workshop. All participants were medical students from the third to the ninth semester (mean 4.31, SD 1.87) at HHU. Of the 26 students, 16 (62%) were in their third semester, 4 (15%) were in their fifth semester, 5 (19%) were in their seventh semester, and 1 (4%) was in their ninth semester (median third semester). All (26/26, 100%) participants attended on all 5 days of the workshop and completed the course with a group presentation of their app concepts and prototypes (ie, there were no dropouts). The participants gave permission to use their intellectual work and feedback for research and publication purposes.

### Common Themes: Narrative Insights From the Group Discussions and Group Work on the App Development

The common themes described in Textbox 2 have been derived either directly from the prototypes or have been identified in plenary discussions. Of the 5 groups, 4 (80%) did not include specific features for medical students in their designs. The main reason for this, the students stated, was that they did not want to be seen as an exclusive target group but rather as students in general. However, they implemented some aspects that are typical of students or people working in health care (eg, shift work). Especially, customizations addressing students’ needs in general (eg, low income, high workload, and irregular schedule) were considered essential. Accordingly, it was important to the students that their app should be provided to university students free of cost. Most groups also offered a variety of ways to customize the app. For instance, push notifications could be scheduled to match users’ preferences or be completely turned off. These customizations were believed to provide a more pleasant user experience and facilitate the daily use of the mHealth app, which was seen as a prerequisite for its success. Moreover, the students considered it important that their app should be easy and intuitive to use for a broad range of users, a reason for this being that medical students have a comprehensive schedule and are not willing to invest a lot of time getting acquainted with an mHealth app. All prototypes included some elements of gamification. In all, 2 aspects stood out because they were repeatedly highlighted by all the groups: data security and evidence base. The students in the workshop considered mental health to be a sensitive topic that should be treated confidentially. The students also seemed to have concerns regarding *big data*: they generally did not approve of companies storing or even selling their data and considered this to be a *no-go area* for an mHealth app. Furthermore, they stated that they would only trust an mHealth app that had been tested and approved by a trustworthy source (eg, their university) and was supported by scientific research.

The students also discussed strategies to improve the uptake of novel e–mental health services such as advertisements using testimonials, including potential negative effects of testimonials. They expressed skepticism regarding such testimonials, especially when they exclusively involve positive ratings. Rather, they preferred balanced reviews (including positive and negative aspects) by trusted sources. Taken together, this points to the need to include user target groups in the design process of mHealth apps to increase their acceptance and use.

**Common themes among all groups during prototyping.**

**Common themes**

- On the basis of scientific evidence
- Certification
- Transparent quality criteria
- Free of cost or cost reimbursement
- Personalization
- Gamification (limited and not too playful)
- Easy and intuitive handling
- Daily use or daily commitment (eg, reminders)

### Case Illustrations: App Development

In this section, the hypothetical prototypes developed by 40% (2/5) of the groups will be presented as exemplary concepts. The 2 prototypes in this paper were chosen based on their visual clarity and comprehensive concept. All creative theoretical work and design samples belong to the students and cannot be used without their permission.

**Figure 1:**
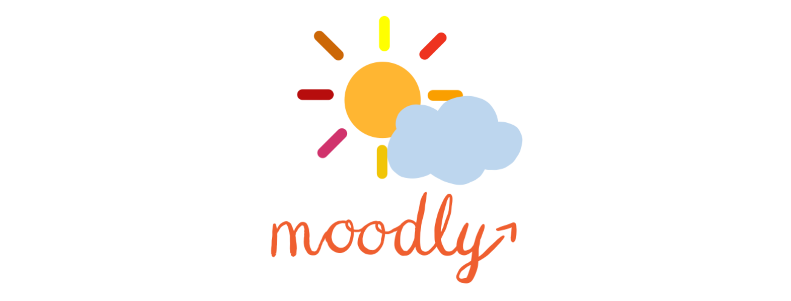
Logo of the app *Moodly*.

**Figure 2:**
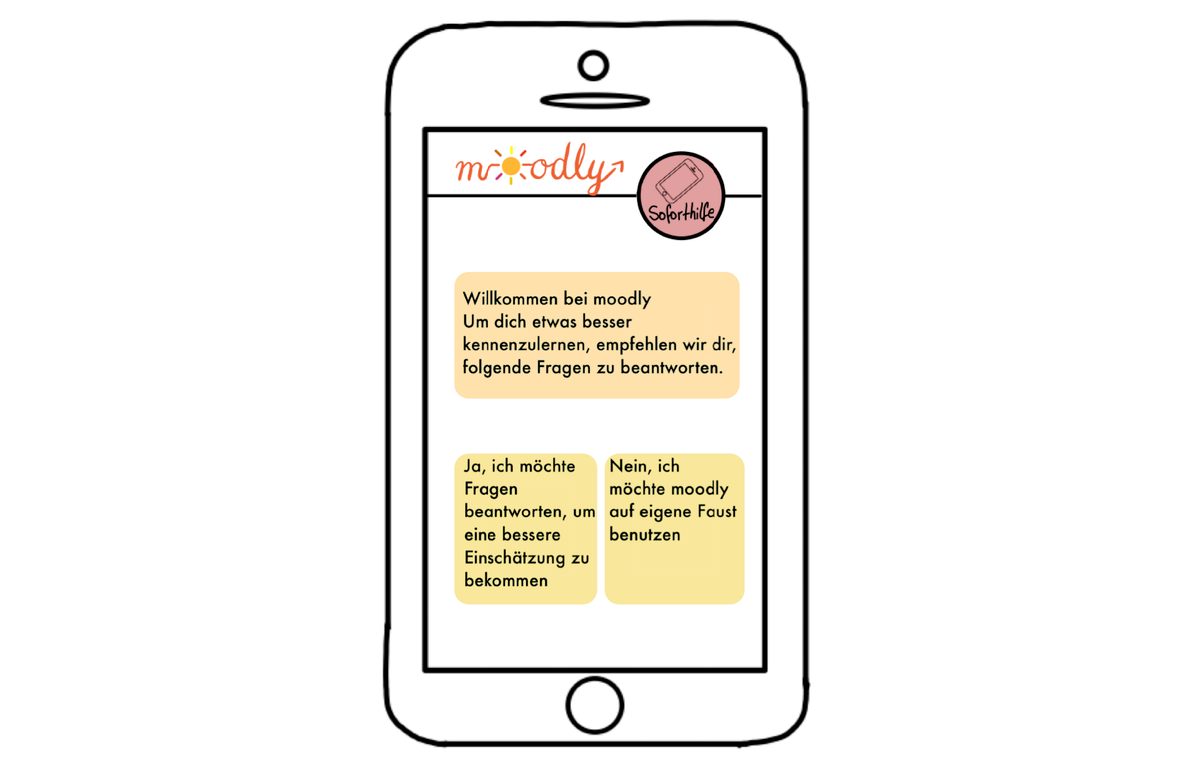
Mock-up of the app *Moodly*: users are asked whether they want to answer questions to personalize the app.

**Figure 3:**
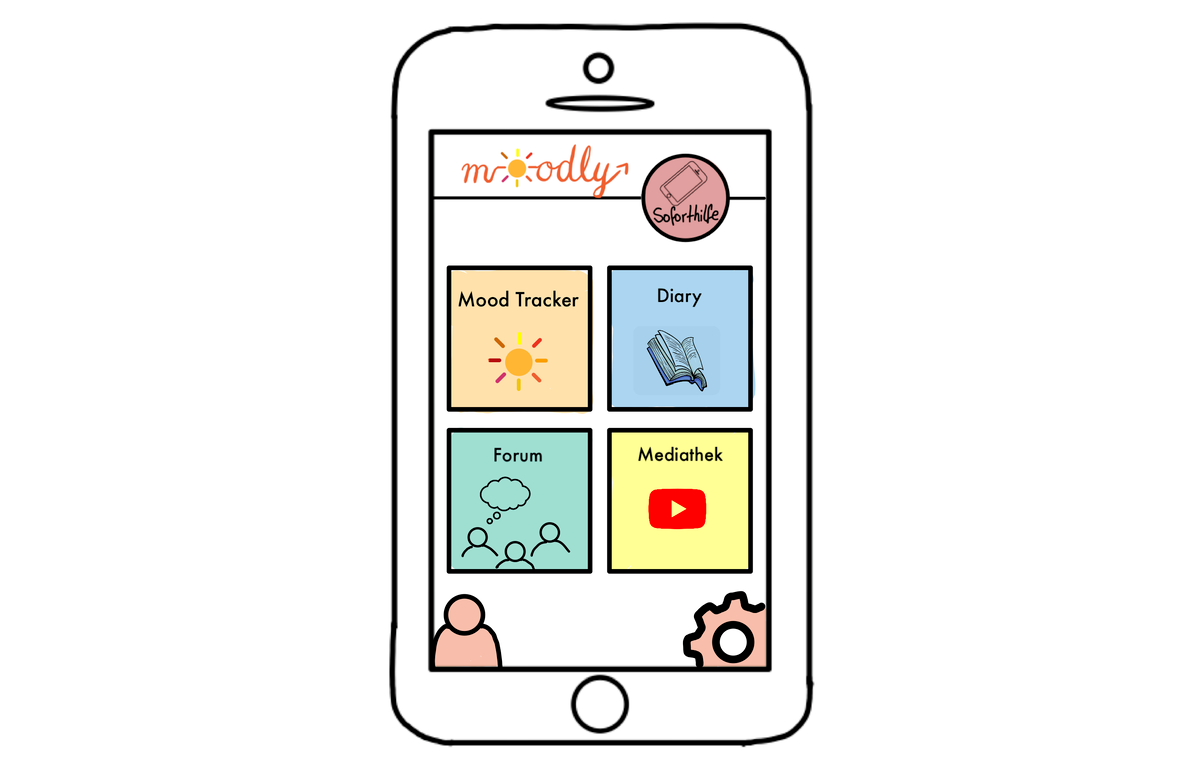
Mock-up of the app *Moodly*: menu and home screen.

**Figure 4:**
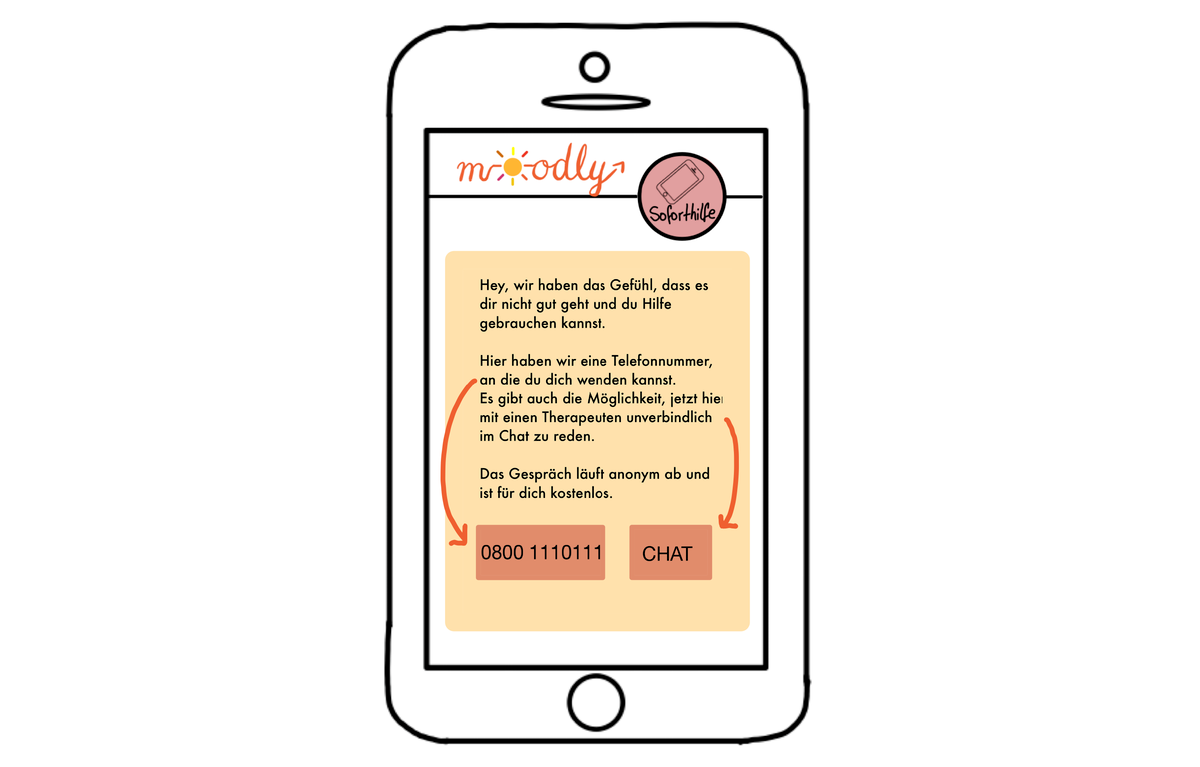
Mock-up of the app *Moodly*: emergency help screen.

**Figure 5:**
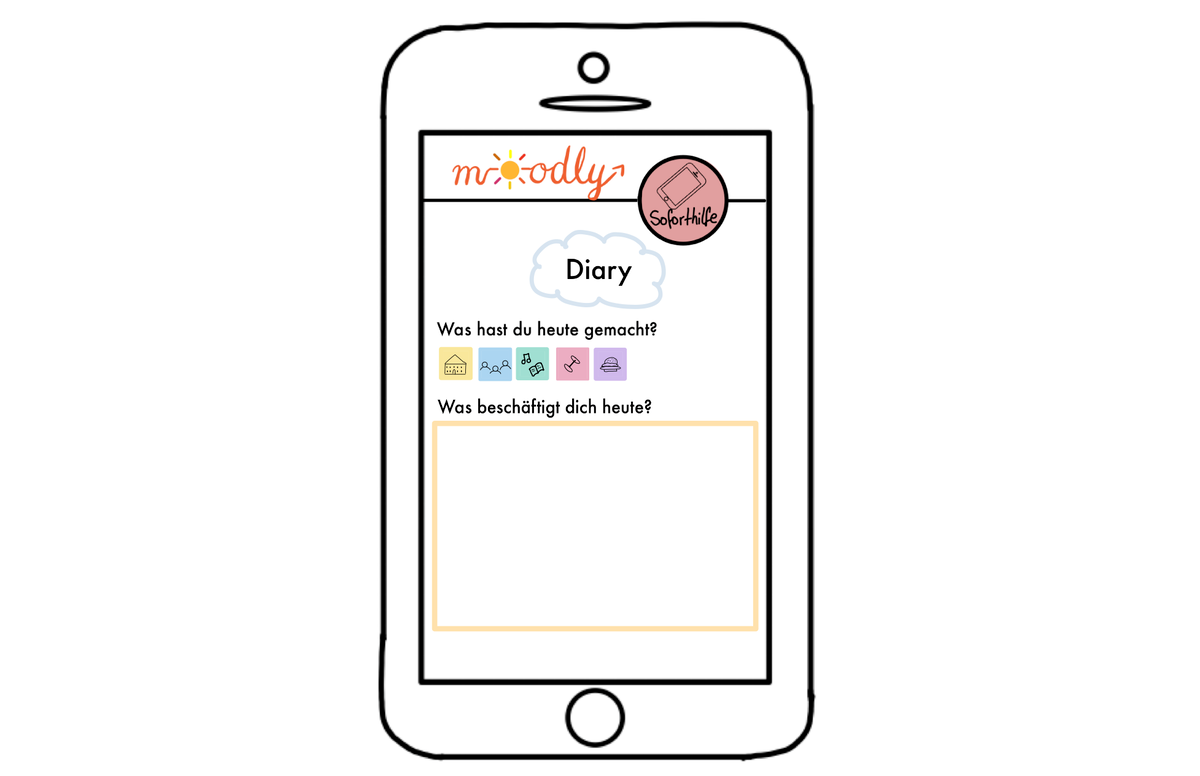
Mock-up of the app *Moodly*: diary.

**Figure 6:**
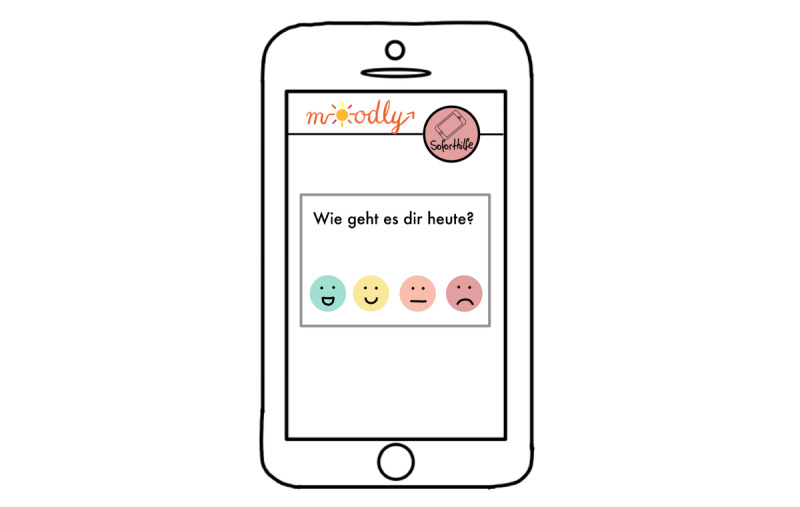
Mock-up of the app *Moodly*: mood rating.

**Figure 7:**
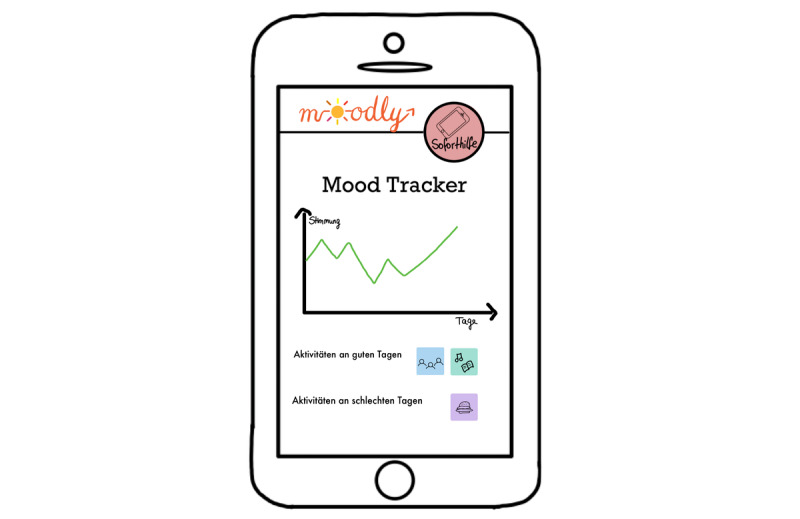
Mock-up of the app *Moodly*: mood tracker.

- *Moodly* (an mHealth app for depressive symptoms [early intervention]; Figures 1-7): a group consisting of 7 students (n=4, 57% were women, and n=3, 43% were men) created the app concept for *Moodly* for mild to moderate depressive symptoms. In Textbox 3, the app is described in the form it would have taken had it been programmed and implemented.

**App concept for *Moodly*.**

***Moodly*: app concept**

- *Moodly* would be accessible as both mobile app and web-based program. The students stated that the goal of the app is to decrease depressive symptoms, impart positive impulses for the day (eg, recommendations for positive activities), and provide guidance to better deal with negative emotions and thoughts (eg, using relaxation exercises). Moreover, it aims to give users a daily structure, improve self-efficacy, and create awareness of their emotions and thoughts.
- The target group consists of not only medical students but also students in general who display depressive symptoms or are experiencing a mild depressive episode (ie, early intervention as the first step or additional support). Users are encouraged to seek professional support; the app informs them that it is not a substitute for treatment.
- The app includes elements of gamification, including personalization, reminders to use the diary, and motivating messages. To increase adherence and use in the long run, users can adapt the app design to their needs and preferences. They can use emojis, upload a picture, or include Graphics Interchange Format files and stickers when making a diary entry. Moreover, the app is structured in a specific order. When students complete a level, they receive a *level up* notification (progress and rewards).
- The students described the design as colorful and esthetic. Users can create a profile and chat and interact with other users. Furthermore, the app provides a variety of helpful videos and resources, for example, to deliver psychoeducation. Therapists and scientists will verify all content in the app. The students highlighted that the app is easy and intuitive to use.
- As the students were worried about people who use the app having an acute mental health crisis, an *emergency help* button is included in every screen (Figures 2-7). If users tap the button, a screen opens through which users can directly contact the German suicide prevention hotline or chat with a psychologist from the app itself (Figure 4). These psychologists are professionally trained in first-line psychotherapy approaches such as cognitive behavioral therapy.
- Before the app is launched in app stores, all students from Heinrich Heine University Düsseldorf would have the opportunity to test it. If they approve it, this can be extended to other universities. Finally, students could provide testimonials for the app in diverse app stores. The entire process would be monitored and assessed by scientists at Heinrich Heine University Düsseldorf.
- Another major concern highlighted by the group presenting *Moodly* was data security. *Moodly* would be strictly anonymous; only admins and therapists available on the app would have access to a user’s email address in case of emergency (eg, suicidal thoughts). Terms of use would be communicated transparently and be easy to understand.
- The app should be used daily.

- *Dreamy Pug* (an mHealth app for insomnia; Figures 8-19): a group of 3 students (n=2, 67% were women, and n=1, 33% was a man) developed the concept for the insomnia app *Dreamy Pug*. In Textbox 4, the app is described in the form it would have taken had it been programmed and implemented.

**Figure 8:**
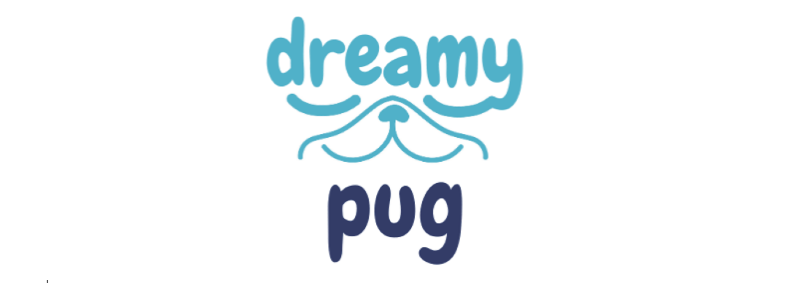
Logo of the app *Dreamy Pug*.

**App concept for *Dreamy Pug*.**

***Dreamy Pug*: app concept**

- The students designed this mobile app for people with insomnia who want to improve their sleep quality.
- Before the first use, users can answer questions (eg, about their age; Figure 15) to receive tailored suggestions on sleep behavior.
- The app is not specially targeted at medical students but rather created for people working in shifts (eg, hospital staff) as they face additional challenges because of their irregular sleep schedule. The app is also suitable for students who often study and sleep in the same place (studio apartment) and face multiple distractions because of their extensive use of smartphones and other electronic devices.
- In case of severe insomnia, users receive an alert to seek help from a professional.
- *Dreamy Pug* aims to increase sleep duration and quality. Moreover, it offers psychoeducation to improve users’ knowledge and understanding of insomnia and the lifestyle and health behavior factors that influence it. This includes exercises to help users fall asleep or for relaxation (eg, progressive muscle relaxation) as well as tips for better sleep hygiene and environment control. All exercises and tips in the app are based on scientific evidence and reflect guidelines from medical societies.
- The avatar *Dreamy the Pug* guides users through the app, explaining its functions. Handling and language of the app have been made as understandable as possible. The app monitors duration and quality of sleep. These data then help to personalize the app to users’ needs and habits.
- Wearables can be connected to the app to improve the quality of sleep tracking. If a user wakes up during sleep, the app recognizes this and immediately offers them help to go back to sleep. In addition, the app can restrict the use of other apps during the time users want to sleep (eg, social media apps). The students stated from personal experience that when they use these apps during the night, going back to sleep becomes harder.
- Further personalization of the app is possible, such as the regulation of push notifications. The app has 3 main modes for night, morning, and day (Figures 11-13). Design and luminosity vary within these modes.
- The group that designed *Dreamy Pug* pointed out that it would be provided free of cost to students after it has been certified and tested in scientific trials.
- The app includes several elements of gamification. The mechanism behind points and rewards is positive reinforcement. If users sleep well, they gain points that they can use to unlock new characters (eg, *Sleepy Fox*) or new sets of blankets for *Dreamy the Pug*.
- Users can also track their progress (Figure 19). In this section, statistics on sleep duration are depicted.
- The app should be used on a daily basis to ensure a reliable sleep profile.

**Figure 9:**
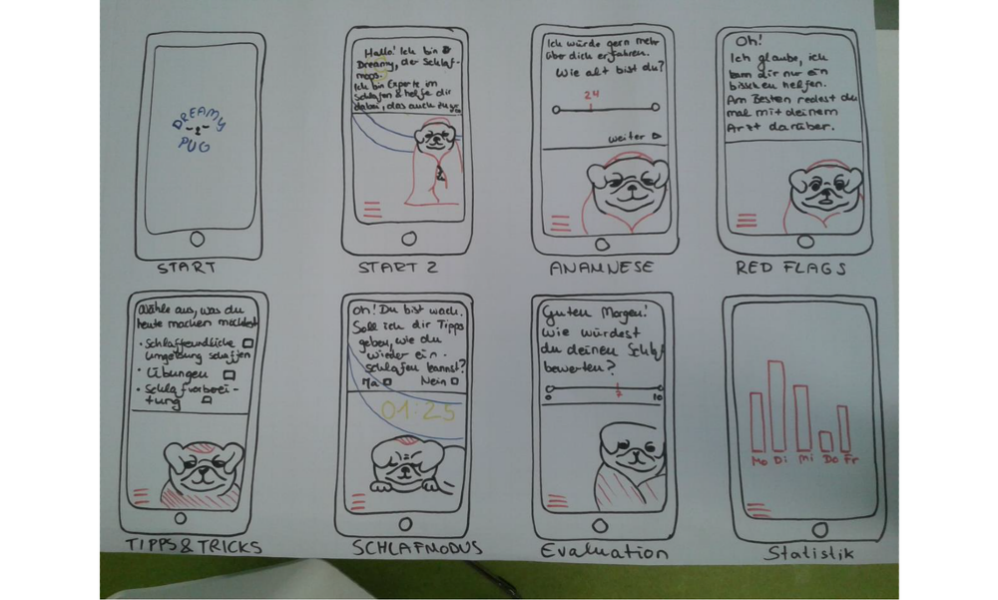
Mock-up of the app *Dreamy Pug*: storyboarding and first draft.

**Figure 10:**
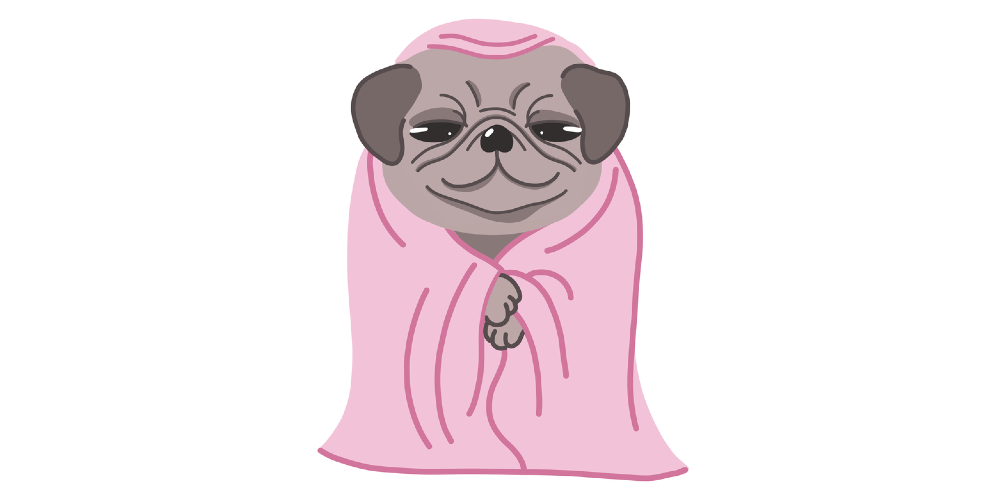
Mock-up of the app *Dreamy Pug*: Dreamy the Pug, the avatar.

**Figure 11:**
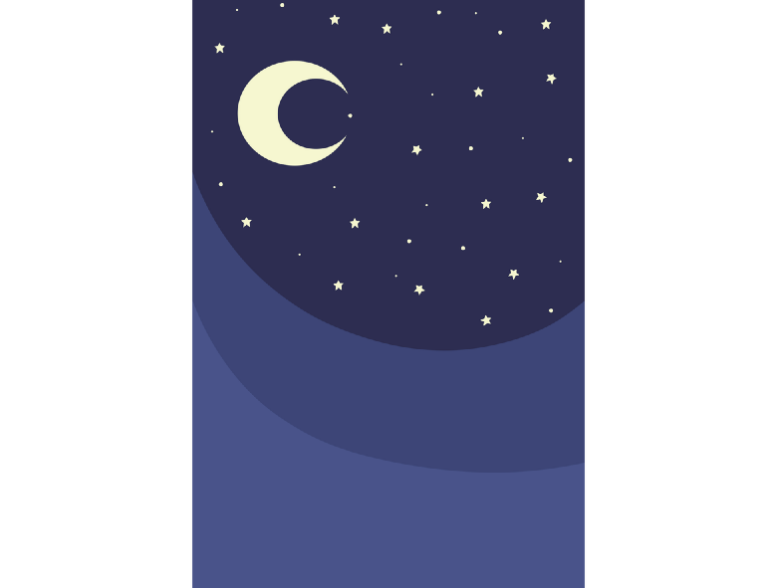
Mock-up of the app *Dreamy Pug*: Screen adapts to different times of the day (here: Night screen).

**Figure 12:**
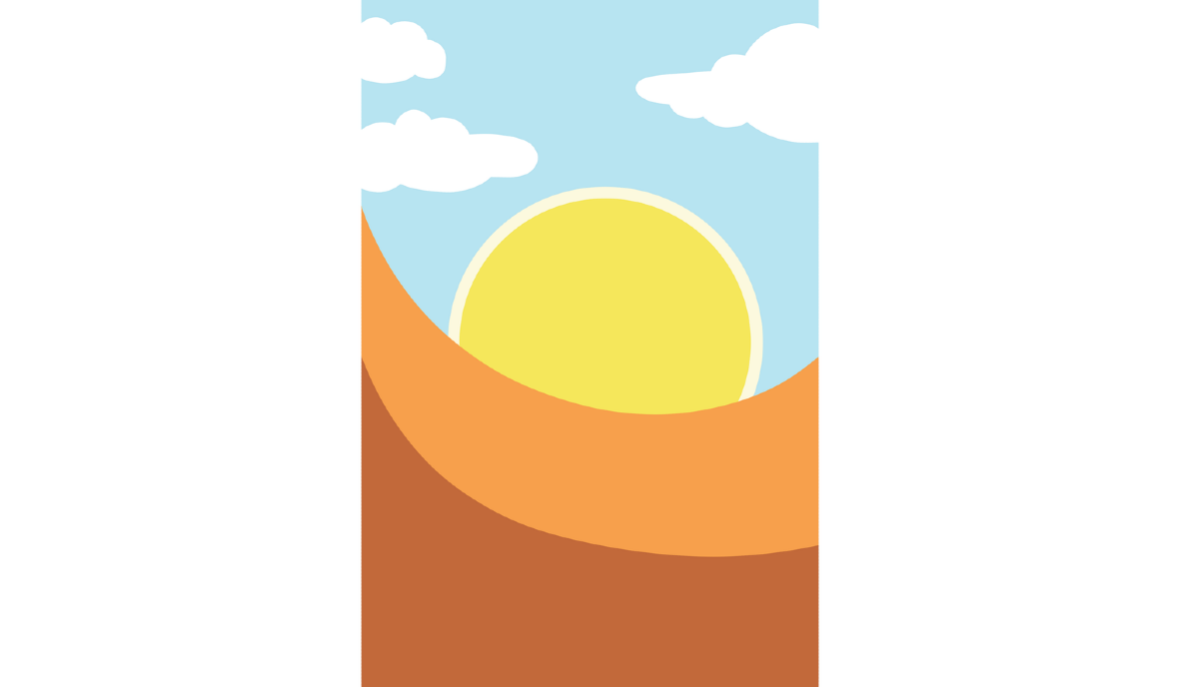
Mock-up of the app *Dreamy Pug*: Screen adapts to different times of the day (here: Morning screen).

**Figure 13:**
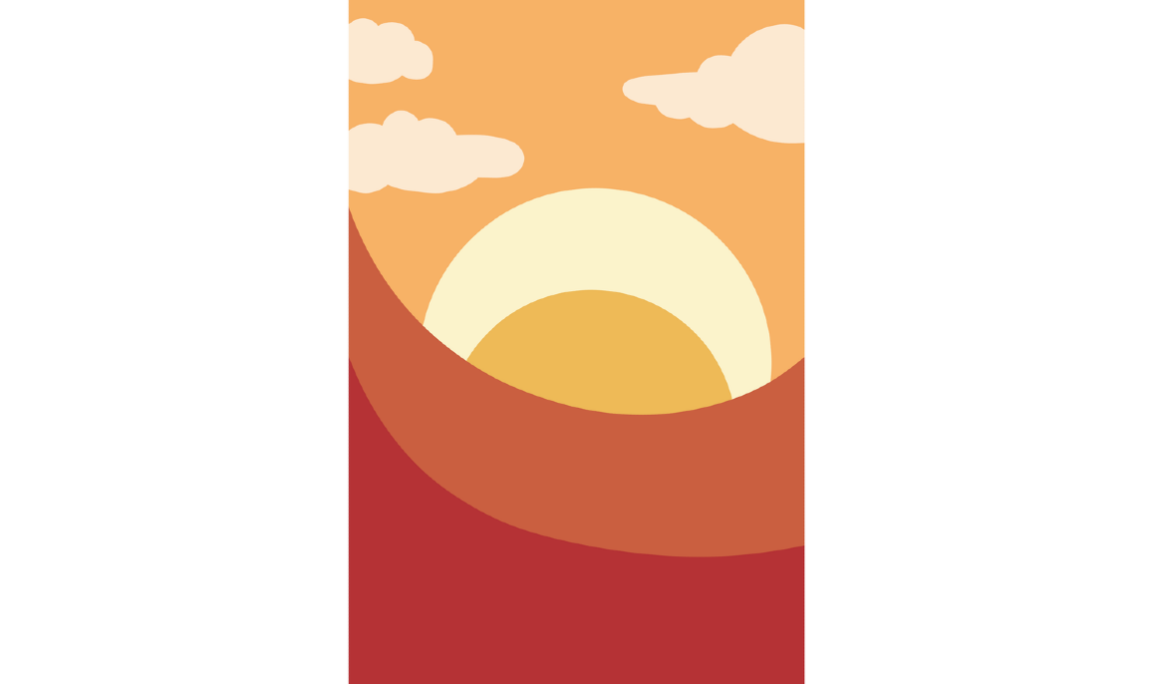
Mock-up of the app *Dreamy Pug*: Screen adapts to different times of the day (here: Evening screen).

**Figure 14:**
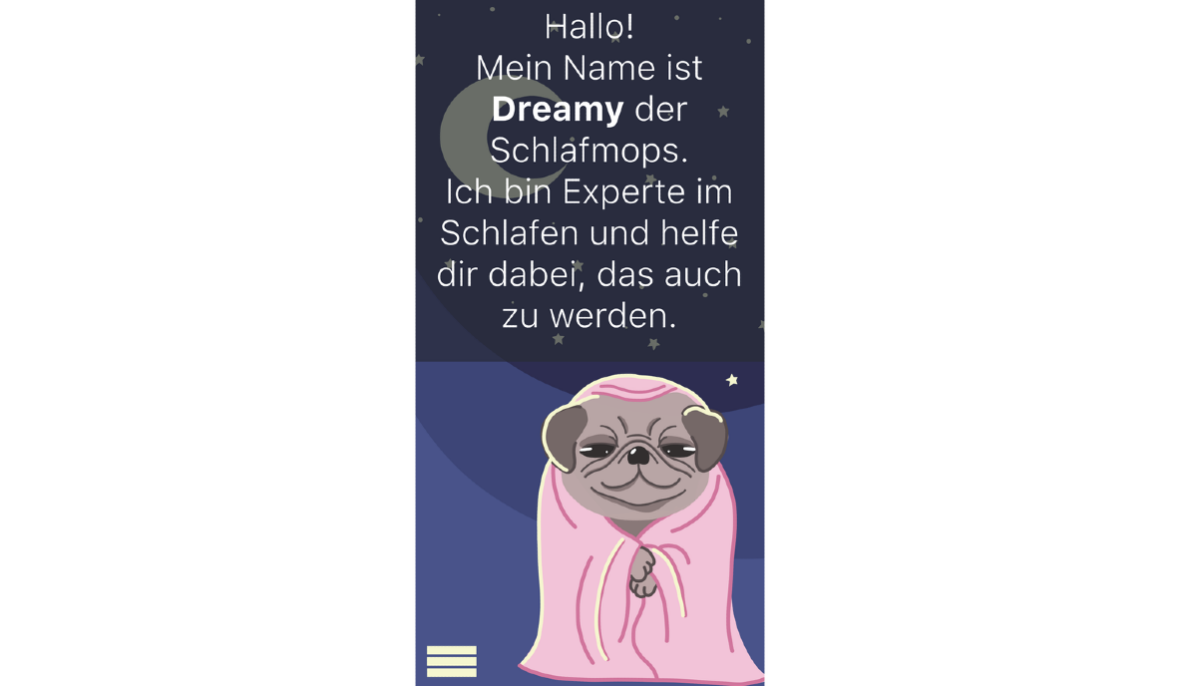
Mock-up of the app *Dreamy Pug*: welcome screen.

**Figure 15:**
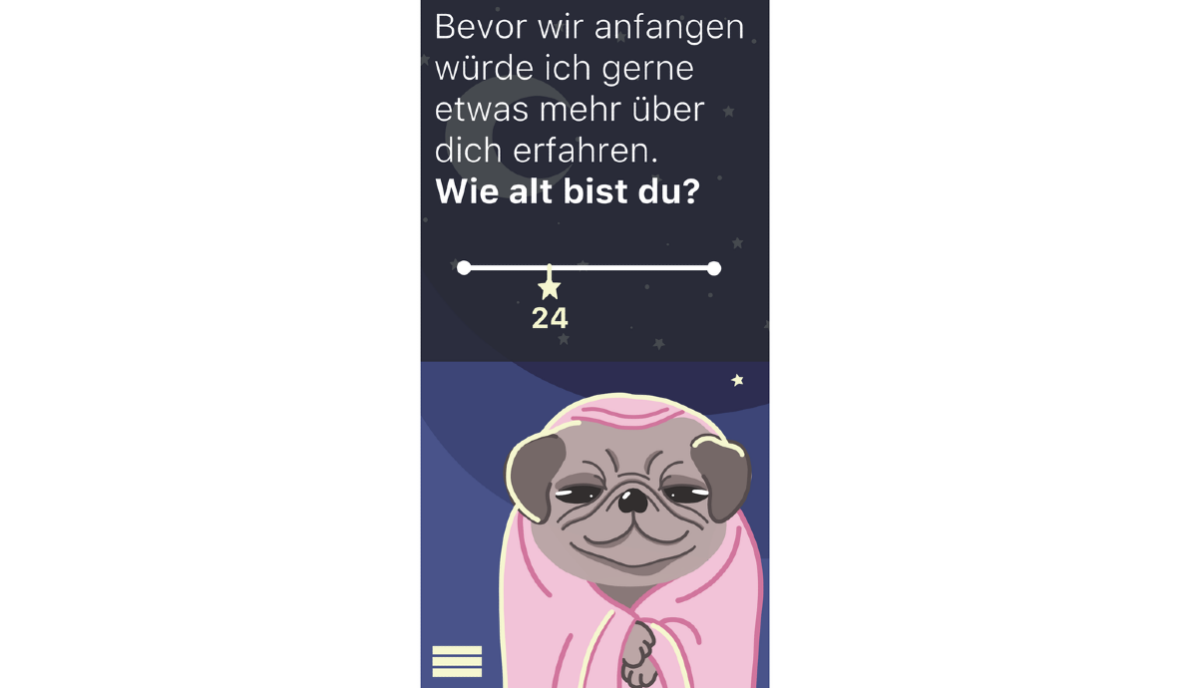
Mock-up of the app *Dreamy Pug*: assessment of personal data (here: age).

**Figure 16:**
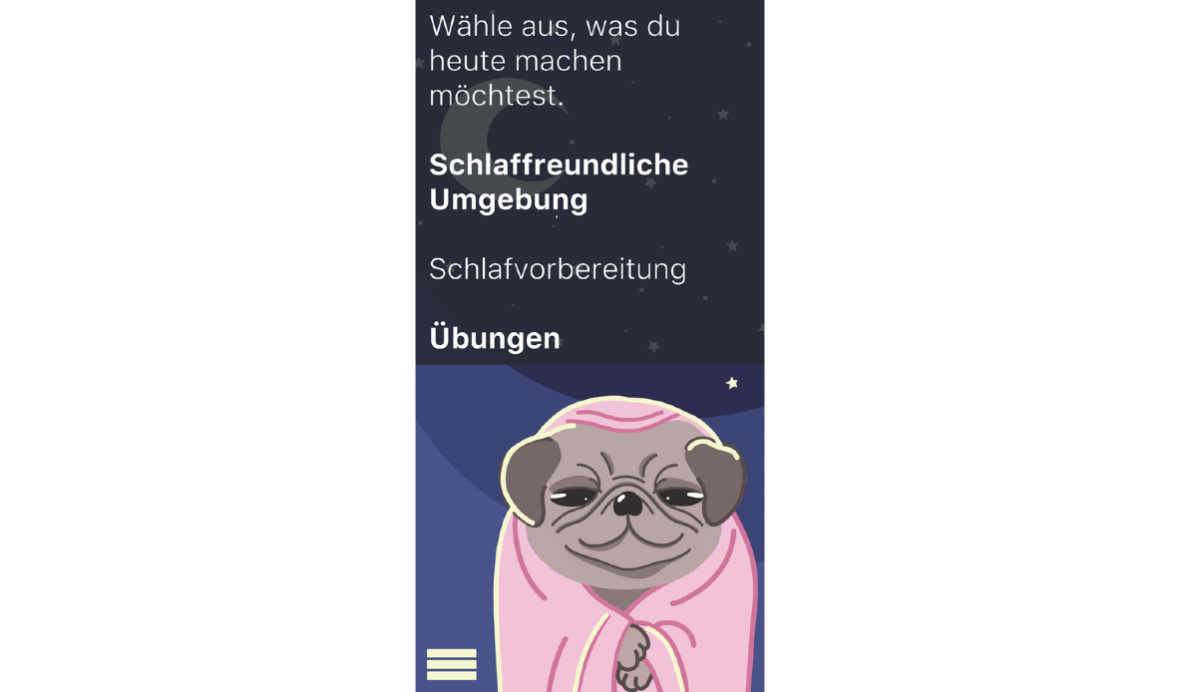
Mock-up of the app *Dreamy Pug*: menu from which to choose different exercises.

**Figure 17:**
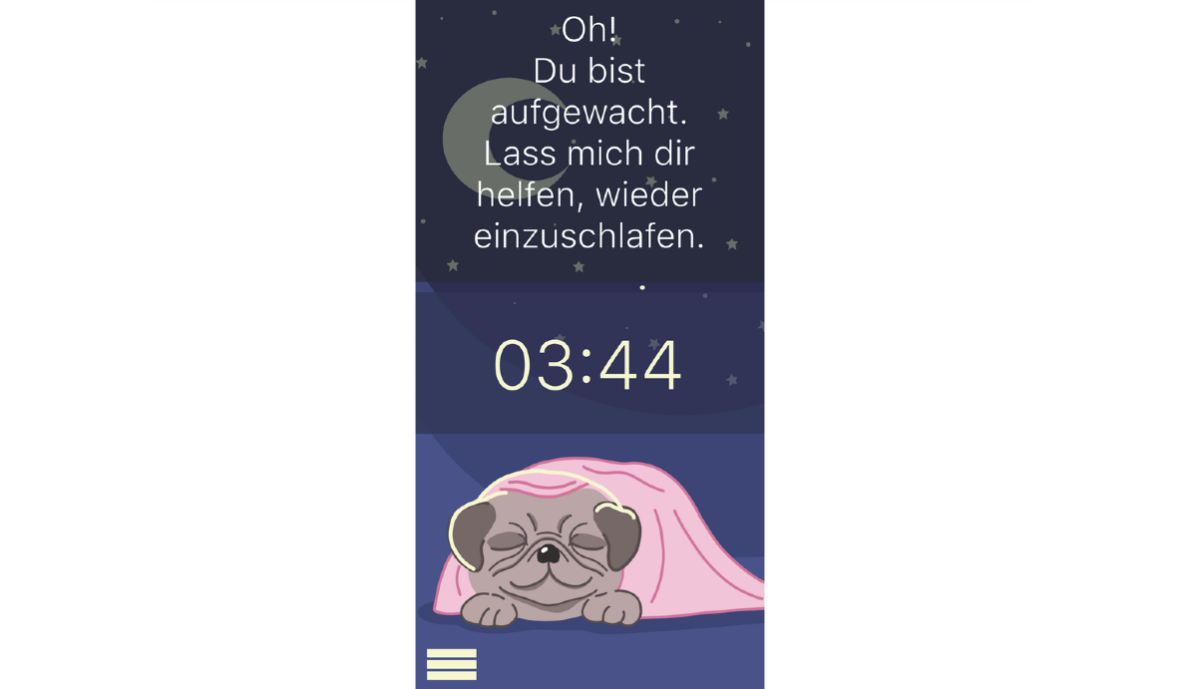
Mock-up of the app *Dreamy Pug*: nightly intervention after user woke up during sleep.

**Figure 18:**
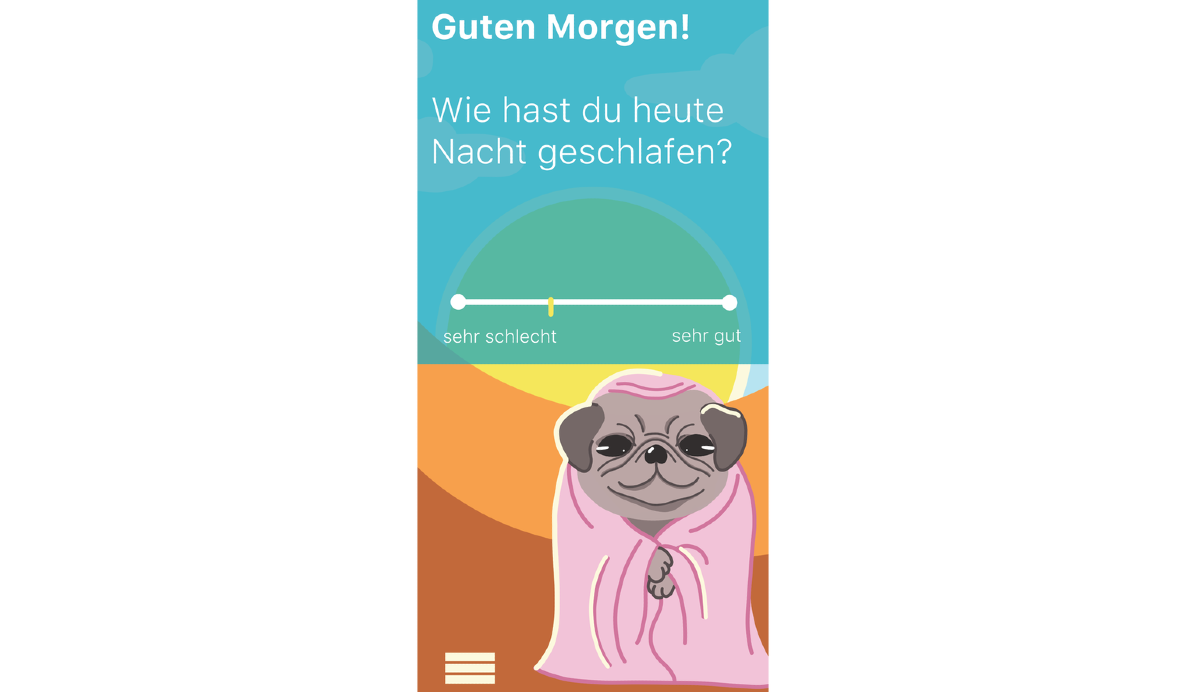
Mock-up of the app *Dreamy Pug*: sleep rating.

**Figure 19:**
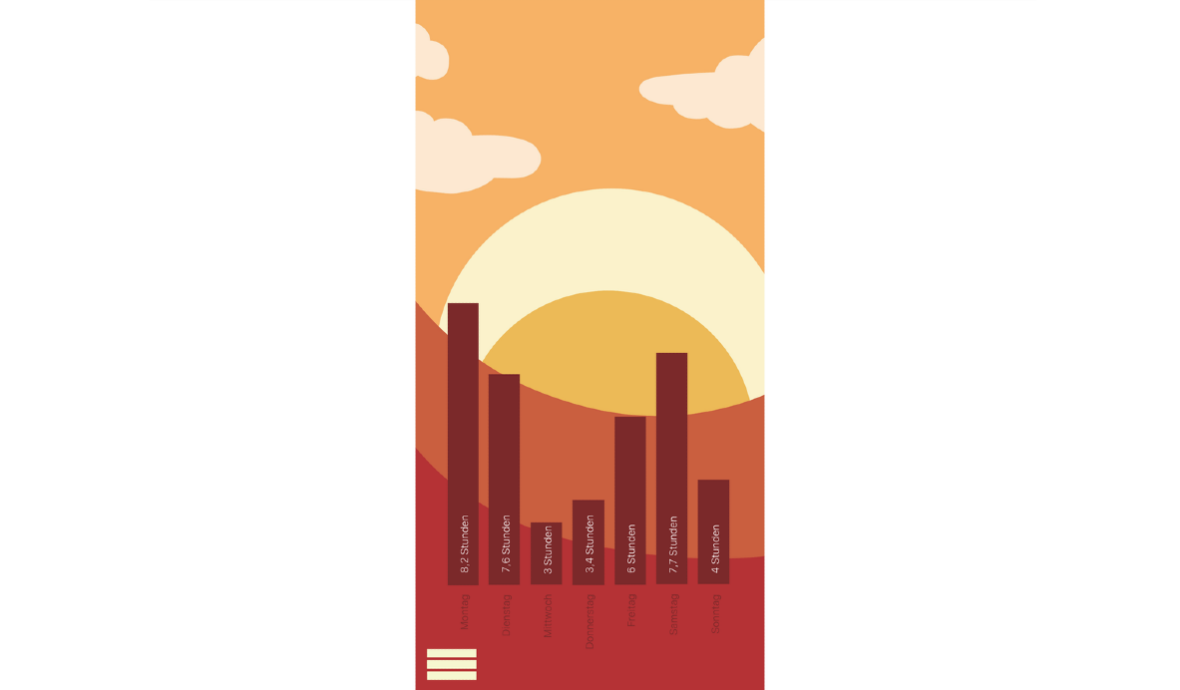
Mock-up of the app *Dreamy Pug*: sleep tracking.

### Workshop Evaluation and Suggestions for Improvement

The feedback from participants upon workshop completion showed that, among other things, it was well-received. The students showed great interest in the presented contents, including the acquisition of knowledge about quality-approved e–mental health solutions. In addition, the students emphasized the benefits of involving potential users in app development. However, the face-to-face workshop was seen to be in need of improvement. The students requested more self-learning components and web-based tools as well as more opportunities to test e–mental health solutions. Moreover, they suggested including more group work, individual work, and interactions in terms of mutual exchange of ideas and exercises of practical relevance. By expanding the digital elective components, the elective course could also be better integrated into everyday life and thus increase the learning effect (self-directed learning and e-learning).

The feedback questionnaire suggests that most of the students were not familiar with e–mental health before the workshop (Table 2). Of the 26 students, 22 (85%) somewhat disagreed, disagreed, or strongly disagreed that they already knew a lot about the learning contents provided at the workshop (mean 2.38, SD 1.10; equals *somewhat disagree*). Of the 26 students, only 1 (4%) agreed that they were familiar with e–mental health before the workshop; 21 (81%) somewhat agreed, agreed, or strongly agreed that they had learned many new things (mean 4.58, SD 1.10; equals *agree*); and 17 (65%) somewhat agreed, agreed, or strongly agreed that they had learned valuable content for their future practice as physicians (mean 3.77, SD 1.24; equals *somewhat agree*).

Students suggested the following improvements in the free-text questions of the feedback questionnaire: learning more about existing apps, testing specific apps, and learning more about app design and technical implementation (“What is needed to create a good app?”).

**Table 2:** Workshop evaluation (scale from 1=strongly disagree to 6=strongly agree).

Question	Values, mean (SD)	Values, median (IQR)
“Before the workshop, I already knew a lot about the topics covered in the workshop”	2.38 (1.10)	2 (1.25)
“I learned a lot of new things in the workshop”	4.58 (1.10)	4.5 (2)
“I have learned a lot of useful things for my profession”	3.77 (1.24)	4 (2)

## Discussion

### Principal Findings

The aim of this study is to describe an innovative concept for an educative workshop following a participatory co-design approach. Moreover, we wanted to present samples of medical students’ prototypes and ideas for mHealth apps, highlighting their preferences and needs.

The co-design workshop was well-received by medical students. It created an environment that allowed participants to engage and be creative from both user and prospective health care provider perspective. The small groups ensured that all participants were able to engage in the design process, as suggested in previous research [40,41].

Moreover, the students provided insights into how mHealth apps can be designed to meet their needs: they agreed that it would be beneficial if the app considered challenges that are specific for shift workers and students in general. Throughout all groups, one of the main issues highlighted by participants was data security provided by their app. This finding fits into the international literature, where data protection concerns have been identified as a key barrier for the adoption of eHealth across various target groups [42].

Confidentiality was another related topic relevant for medical students, especially because many mental health apps do not fulfill this criterion [43,44]. This is interesting to note, considering that stigmatization of mental illness is still prevalent in the medical field, and especially students fear professional disadvantages from the disclosure of a mental health problem [2,45]. It is important that mHealth apps provided for medical students are approved by trustworthy sources such as the students’ university [27]. Similarly, students perceived the app being tested in scientific trials and receiving certification for its effectiveness as an indicator of app quality. Furthermore, the students suggested that information regarding the evidence base, including references to randomized controlled trials, should be integrated into the app’s description to support informed decisions. Further information on the provider or on data security should be included in the app’s terms and conditions section.

In addition, the participants declared customizable elements, easy and flexible use, and daily commitment as essential during the presentation of their mHealth app prototypes. This is in line with the principles of persuasive design, which has also gained importance in the health informatics educational sector in recent years [46]. All groups included elements of gamification in their apps, which is in line with the determinant hedonic motivation in the UTAUT2 model [33] and aims to foster user engagement, motivation, and adherence [47]. Moreover, gamification might improve the learning process in health education [48].

However, the students were critical of parts of the lectures on mHealth apps because they had difficulty following them on an abstract level without an mHealth app for practical demonstration. This was due to the limited availability of freely accessible quality-approved mHealth apps, which has been acknowledged as a barrier in previous mHealth educational research with medical students [24]. Hence, future educational workshops should preselect suitable mHealth apps and provide them to students in the workshop.

Interestingly, the group discussions and prototypes developed in this workshop suggest that most medical students do not see an urgent need for mHealth apps directly targeted at medical students [27]. Only specific features for students (eg, low income, high workload, and exam-related distress) or people working in health care (eg, shift work) were proposed.

Several quality-approved, effective digital mental health interventions for students exist [5,49], but they are not well known or used by medical students. Therefore, the key challenge is to reach medical students. This could be achieved with targeted information or through specific channels (eg, student support groups and mentoring [49]). However, medical students may not be particularly interested in apps that are exclusively designed for them; rather, they might be interested in apps that are designed for students in general. Medical students are at high risk for certain mental illnesses [30,50,51], and their lack of help-seeking behavior is of great concern [52]. Thus, it is important to increase the adoption of psychological services, including early interventions, for example, by offering mental health apps. Overall, medical students in our workshops preferred to emphasize similarities rather than diverging factors between them and other student groups. However, to be able to answer the question of whether mHealth apps targeted at medical students are desired, a more representative sample is required.

Another goal of the workshop was knowledge acquisition and transfer in terms of competence-based collaborative learning (ie, application of acquired knowledge).

Overall, it is striking that only 31% (8/26) of the students agreed or strongly agreed that they had learned anything they consider valuable for their future practice. It is possible that the students did not deem eHealth relevant for their profession, which hints at the need for more education to familiarize students with eHealth. Generally, this finding can be seen in light of the period when the workshop was conducted, namely in early March 2020, 1 week before the first lockdown due to the COVID-19 pandemic in Germany. The pandemic was an unexpected driver for the transition of telemedicine into health care, especially the spread of videoconferencing consultations and also e–mental health services [53].

Furthermore, it is possible that some contents such as the demonstration of the UTAUT2 model [33] were too theoretical and not directly transferrable to clinical practice. On the basis of the students’ feedback, we have revised the workshop contents and format to integrate more practically relevant topics (eg, the concrete procedure for the prescription of apps and legal issues) and to tailor the workshop to individual needs and preferences (see *Lessons Learned* section). Overall, more efforts are needed to implement suitable educational workshops on the digitalization of health care in the medical curriculum, especially considering that increasingly more medical students recognize the relevance of the topic for their future profession [54,55].

### Lessons Learned

The suggestions received at this pilot workshop have been transferred to a novel e-learning participatory design workshop with medical students. It was conducted for the first time in the 2021 summer semester (e–mental health literacy as an elective subject, with support of the Medical Faculty Quality Funds for innovative educational projects). A novelty of the new e-learning workshop on e–mental health and mHealth for medical students is that it offers an extensive e-learning module on the quality criteria of mHealth apps as well as new ways of implementing remote group work (under continuous guidance by the team of educators), and their integration into routine care, design thinking, and gamification. This helps to systematically guide small groups of medical students through the design of a prototype mHealth app, alongside engaging sessions and continuous tailored feedback. The platforms used are ILIAS (an open-source digital learning platform for asynchronous self-directed learning), Webex (Cisco Systems, Inc), and Microsoft Teams (videoconference-based *live* synchronous meetings and collaborative learning), as well as free prototyping software. On the basis of the students’ feedback, the workshop will be iteratively refined using participatory design approaches.

Thus, the subsequent workshop included new educational content of more practical relevance for medical students, such as the prescription of apps. Moreover, the switch to e-learning seems to have facilitated knowledge acquisition significantly: the second workshop was evaluated more positively compared with the pilot workshop, and knowledge acquisition was rated consistently as *high*. Future workshops could also include objective tests regarding the improvement of eHealth literacy, for example, through a quiz at the start and the end of the workshop (pre–post design). However, for us, it was primarily important to learn whether students deem the workshops to be valuable as an educational tool and which aspects need to be adopted for future workshops.

Upon completion of the quality-improvement project, the participatory workshop will be implemented as a standard elective subject in the medical curriculum at HHU. The curriculum will likely be extended to other fields in eHealth as well, such as digital health for chronic conditions. Moreover, a collaboration with the university’s computer science department is planned that could offer the opportunity to translate medical students’ ideas into actual mHealth apps. This case study lays the foundation for these ambitions by exploring medical students’ perspective in detail, providing concept sketches, and initiating communication channels.

### Limitations

The exploratory nature of our case report entails several limitations that must be considered. First, our results concerning medical students’ ideas and preferences for mHealth apps are not conclusively generalizable to the entire medical student population. Rather, this case study offers subjective insights into participatory workshops for educational purposes from the educators’ perspective.

A second concern related to generalizability is that participants chose the workshop as an elective course. Thus, there might be a self-selection bias if especially students familiar with, or interested in, mHealth attended the workshop. Students with no interest in mHealth could have had other ideas or preferences compared with those of the workshop participants. However, the students indicated low familiarity with eHealth, and many chose the workshop as an elective course because it was held on 5 consecutive days during the semester holidays.

Third, all participants were regular smartphone users as well as digital natives and therefore widely familiar with smartphone apps. It became evident on different occasions (eg, in group discussions and feedback rounds) that they had already formed critical opinions on related topics (eg, *big data*) before the workshop. This might be why they highlighted the importance of data security.

Furthermore, it is likely that the different lectures, exercises, and tasks throughout the workshop (eg, creating avatars and personas as well as learning about mHealth guidelines) influenced the students’ prototypes. However, they only included elements that they perceived as useful for their prototype (eg, no avatar in *Moodly*).

Finally, the workshop was conducted in Germany where digital health is not yet a mandatory or widespread part of medical education [22]. However, it is important to note that medical schools in many countries worldwide have already recognized the urgent need to implement eHealth in the curriculum. The rate of progress in the digitalization of health care has increased, especially since 2020, for instance, in Switzerland [56]. A next step for German medical schools would be to integrate digital competencies in the NKLM (*Nationaler Kompetenzbasierter Lernzielkatalog Medizin* or National Competence-Based Learning Goal Catalog for Medicine) accordingly [22,57].

### Implications and Recommendations

#### For Researchers

Researchers might consider the following implications and recommendations:

- Develop an empirical and theory-led guide for best practice through continuous evaluation of medical students’ preferences and needs using both qualitative and quantitative research methods.
- Define and test outcomes of the learning success by combining subjective and objective measures based on digital health technology literacy frameworks [58]. However, note that there is a lack of randomized controlled trials in the field because workshops within elective subjects pose organizational and ethical challenges for this particular study design [59].
- Enable the cocreation of educational content using participatory research approaches (eg, person-based approach [60]).

#### For Lecturers

Lecturers might consider the following implications and recommendations:

- Define a set of clear competencies and learning goals that should be obtained through the workshop [55].
- Provide personalized and interactive digital learning platforms in line with recent trends [55].
- Select and use novel educational tools and web-based platforms such as Psy-Q [61].
- Learn how to create apps—easy and intuitive software tools to build initial apps exist (eg, iBuildApp [62]).
- Encourage the collaboration of physicians and informatics experts as lecturers, for example, as shown in the DigiWissMed project in Germany [63].
- Offer trainings in digital health for medical educators.

#### For Medical Schools

Medical schools might consider the following implications and recommendations:

- Note that in Germany, most eHealth-related topics are taught within elective subjects, and the number of such courses is very limited [22]. Usually, these existing eHealth courses in Germany do not consider mental health as a relevant topic for medical students as potential users and future physicians. Hence, there is a need not only for eHealth education in general, but also for digital mental health in particular. Surveys could help to determine the needs and preferences of medical students regarding the implementation of eHealth in the curriculum.
- Note that not all medical students may be interested in eHealth or in creating their own apps in the same way. Thus, basic knowledge on eHealth could be implemented in the standard curriculum, whereas more advanced or in-depth courses could continue to be part of the elective curriculum [64].

### Conclusions

Overall, the participatory workshop on e–mental health was well-received by medical students. Thus, it seems to be a feasible approach that can be used as a starting point for future educational activities with medical students. Moreover, the medical students had a clear vision for their ideal mHealth apps after being informed about key quality criteria and persuasive design features. As medical students are both potential users and future health care providers, the adoption of mHealth education into the medical curriculum should be considered.

## Abbreviations

HHU: Heinrich Heine University Düsseldorf
mHealth: mobile health
NKLM: Nationaler Kompetenzbasierter Lernzielkatalog Medizin
UTAUT2: Unified Theory of Acceptance and Use of Technology, extended version
